# Mortality in Recreational Mountain-Biking in the Austrian Alps: A Retrospective Study over 16 Years

**DOI:** 10.3390/ijerph191911965

**Published:** 2022-09-22

**Authors:** Elena Pocecco, Hamed Wafa, Johannes Burtscher, Peter Paal, Peter Plattner, Markus Posch, Gerhard Ruedl

**Affiliations:** 1 Department of Sport Science, University of Innsbruck, 6020 Innsbruck, Austria; 2 Medical University of Innsbruck, 6020 Innsbruck, Austria; 3 Department of Biomedical Sciences, University of Lausanne, 1005 Lausanne, Switzerland; 4 Institute of Sport Sciences, University of Lausanne, 1015 Lausanne, Switzerland; 5 Austrian Board for Mountain Safety, 6020 Innsbruck, Austria; 6 Department of Anaesthesiology and Intensive Care Medicine, Paracelsus Medical University, 5020 Salzburg, Austria

**Keywords:** fatalities, accidents, severe sports injuries, sports epidemiology, injury prevention, bicycling, cycling, mountain sport, outdoor activity, alpine sport

## Abstract

Despite recreational mountain-biking’s growing popularity worldwide, the literature on mortality in this leisure sporting activity is scarce. Therefore, the aim of the present study was to investigate the characteristics of fatal accidents as well as resulting dead victims during recreational mountain-biking in the Austrian Alps over the past 16 years. For this purpose, a retrospective study based on Austrian institutional documentation from 2006 to 2021 was conducted. In total, 97 fatalities (1 woman) with a mean age of 55.6 ± 13.9 years were recorded by the Austrian Alpine Police. Of those, 54.6% died due to a non-traumatic (mostly cardio-vascular) and 41.2% due to a traumatic event. Mountain-bikers fatally accidented for non-traumatic reasons frequently belonged to older age classes (*p* = 0.05) and mostly (73.6%) died during the ascent, whereas traumatic events mainly (70.0%) happened during the descent (*p* < 0.001). Throughout the examined period, the absolute number of fatalities slightly increased, whereas the mortality index (proportion of deaths/accidented victims) did not (mean value: 1.34 ± 0.56%). Factors such as male sex in general, above average age and uphill riding for non-traumatic accidents, as well as downhill riding for traumatic events, seem to be associated with fatalities during recreational mountain-biking in the Austrian Alps. These results should be considered for future preventive strategies in recreational mountain-biking.

## 1. Introduction

Mountain biking (MTB) is characterized by cycling off-road, often over rough terrain, using specially designed bikes [1]. It is an Olympic sport and a very common recreational activity, especially in the mountains during the summer season. In the European Alps, it has recently been estimated that there are approximately 18.7 million mountain bikers (locals and visitors) [2]. The number of practitioners is rising worldwide: e.g., in Germany, it increased by 9.6% from 2014 to 2018 and by a further 10.9% (from 14.9 to 16.6 million people) between 2018 and 2022 [3]. In the US, about 26.1% more mountain-bikers were recorded over a decade: from 6.9 million in 2007 to 8.7 million in 2018 [4]. With about 133,000–146,000 annually sold pieces between 2013 and 2016, mountain-bike is the most sold type of bicycle in Austria [5]. Additionally, the number of sold e-bikes has dramatically increased in Austria in the past 14 years: from ca. 8000 in 2008 to ca. 220,500 in 2021 [6]. Among them, especially the selling of electric mountain-bikes is increasing the most, with about 77,500 sold pieces in 2019, which means an increase of 23% in comparison to 2018 [7]. A representative survey among nearly 15,000 members of the Austrian Alpine Club in 2020 showed that 83% of the respondents practice MTB at least once a year, and 55% of those used their mountain bike more than 20 times per year; overall, 27% of respondents use an e-mountain-bike [8].

Regular cycling has been shown to have health benefits and especially positive effects on chronic diseases, i.e., it was associated with a reduction in cardiovascular risk [9] and with a lower risk of diabetes type 2 [10]. In particular, MTB has the capacity to improve health-related fitness [11], mental health, and well-being [12,13]. Moreover, the whole-body vibration experienced during MTB appears to influence immune responses, at least of elite enduro racers [14], as well as to stimulate osteogenic bone signaling in trained mountain-bikers [15]. However, MTB is also associated with specific risks, such as accidents and dangerous spine injuries [16,17,18]. In addition, extreme MTB challenges might cause sub-clinical myocardial injuries in male amateur mountain-bikers [19].

Despite the considerable risk of fatal and non-fatal accidents during this popular outdoor sporting activity, only a few studies reported data on severe MTB injuries, including fatalities [18,20]. In particular, Kim et al. [20] reviewed serious MTB injuries requiring trauma center admission during 10-year lasting research conducted in the three trauma centers of the Greater Vancouver area. From 1992 to 2002, one patient out of 399 mountain-bikers died in the hospital [20], which corresponds to a case fatality rate of 0.25% (2.5 out of 1000 injured mountain bikers). Considering a total number of estimated 56,000 mountain bikers in the bike park and on the trails of the area served by the three hospitals [20], a mortality rate of approximately 0.0002% (2 out of 1 million mountain bikers) can be estimated. A higher case fatality rate has been presented by Roberts et al. [18]: among 49 severe injured adult mountain-bikers between 1995 and 2009, one subject died, which means a case fatality rate of 2.04% (20.4 out of 1000 severely injured mountain-bikers) over 14 years. Just a couple of studies reviewed the few data on mortality in MTB available in the literature, also presenting spare data from the Austrian Alps [21,22]. However, no research until now seems to focus attention on factors related to mortality in recreational MTB, as it has been conducted in the past for other summer and winter leisure alpine sports [23,24,25].

Therefore, the objective of the present study was to present characteristics of fatal accidents as well as resulting dead victims during recreational mountain-biking in the Austrian Alps during the past 16 years, suggesting possible factors associated with fatal events.

## 2. Materials and Methods

### 2.1. Study Design

The present study was designed as a retrospective analysis of documented accidents in mountain-bikers in Austrian mountains during a 16-year period (2006–2021). It was conducted according to previous studies on fatal accidents in mountain sports such as hiking and skiing/snowboarding in the Austrian Alps [23,24,25].

This study was performed in conformity with the ethical standards of the 2013 version of the Declaration of Helsinki and was approved (approval ID-73/2022) by the institutional review board (IRB) of the Department of Sport Science as well as the Board for Ethical Issues (BfEI) of the University of Innsbruck.

### 2.2. Data Collection and Primary Data Source

The primary data source was the database for accidents and emergencies in the mountains of the Austrian Alpine Police (part of the Austrian Federal Ministry of the Interior, BMI). Instructed staff of the Austrian Alpine Police continuously recorded information on accidents and emergencies, including fatalities during activities in the Austrian mountains in the course of their routine work using standardized software forms. In certain cases (i.e., fatal accidents), information was gained by an onsite inspection of the Austrian Alpine Police staff. Data were processed and stored by the Austrian Board for Alpine Safety (ÖKAS).

The following details of each accident were entered in the database: categorized type of activity during which the accident occurred (here: ‘mountain-biking’), date and time, location of the accident (federal province, district, mountain and route, approximated altitude, and direction of exposition), lighting conditions (‘daylight’, ‘twilight’, and ‘darkness’) and weather conditions at the time of the accident (e.g., ‘sunny’, ‘overcast sky’, ‘raining’, ‘snowing’, ‘foggy’), the number of involved persons/victims, and a short description of the accident’s sequence of events. The following details for each victim were recorded: sex, age, nationality, wearing of a helmet, degree of illness or injury (‘unharmed’, ‘slightly injured’, ‘seriously or life-threatening injured’ or ‘dead’), cause of accident (e.g., ‘cardiovascular disease’, ‘illness’, ‘fall’, ‘collision’, ’getting lost/losing the way‘, ‘other’, ‘unknown’), type of illness or injury (e.g., ‘cardiovascular disease’, ‘fracture’, ‘luxation’, ‘internal injury’, ‘polytrauma’, ‘exhaustion’, ‘unknown’), as well as location of illness or injury (e.g., ‘chest’, ‘head’, ‘neck/cervical spine’, ‘back/spine’, ‘whole body’). Moreover, it was registered whether the accident occurred during the ascent, the descent, or during a different situation, and information on the type of trail (‘asphalt road’, ‘drive or forest way’, ‘marked hiking trail or a small path’, ‘pathless terrain’, ‘bike-park’, ‘other’) were recorded.

In the case of rare answers, these were grouped and classified as ‘other’.

### 2.3. Data Transfer

The Austrian Board of Alpine Safety had access to the database of the Austrian Alpine Police and has approved to use of the anonymized data for analyses. For the present study, the ÖKAS provided a data set including all recorded accidents (non-fatal and fatal), which were categorized as the activity ‘mountain-biking’ in the Austrian Alps during the period 2006 to 2021 to the Department of Sport Science (University of Innsbruck, Austria) for data analysis. This primary data source is comprised of information on a total of 7675 accidents involving 7892 mountain-bikers and includes 97 fatal accidents/deaths.

### 2.4. Data Screening and Data Selection

In the first step, all accidents were independently screened by two authors (EP and HW) for potential exclusion criteria. An exclusion criterion was given when the type of activity at the time of the accident did not meet the definition of recreational mountain-biking, i.e., the accident happened during a professional race. No accidents or mountain-bikers were excluded.

Concerning traumatic fatalities, causes of accidents could be identified and classified by two authors (EP and HW) on the basis of the accident description provided by the Alpine Police. In case there was no unanimous accordance concerning the classification, the opinion of a third expert was asked and was considered binding.

The time of the day was classified in accordance with Roberts et al. [18] (Table 1).

Age classes of 10-year intervals correspond to the literature [8,24]. The mortality index was calculated as the proportion of deaths of fatally and non-fatally accidented victims [24] both per year and as the mean value over the 16-year period.

### 2.5. Statistics

Data are presented descriptively as means ± standard deviations, absolute and relative frequencies, as well as rates. Shapiro–Wilk test was used to check normal distribution, and datasets were compared by *t*-test or Mann–Whitney U-test, as appropriate. Death rates were compared by χ^2^ tests, and regression analyses were performed to detect possible trends over time. Except for the results concerning fatal accidents over time and mortality index, data of the whole observed period of 16 years were combined and analyzed. SPSS 27.0 (IBM Corporation, Armonk, NY, USA) was used for the statistical analysis. *p*-values were two-tailed, and values ≤ 0.05 were considered statistically significant.

## 3. Results

### 3.1. Characteristics of Accidents

Table 1 shows environmental characteristics associated with the deaths of recreational mountain-bikers during the present study. In total, 97 fatal accidents were registered, the highest number thereof in the region of Tyrol (35.1%). Most fatalities (92.7%) happened in the period between May and October, whereat most cases were registered in June (25.8%); they happened during weekdays as well as weekends (60.8% vs. 39.2%, respectively). As 79.4% of lethal accidents happened between 8.00 a.m. and 4.00 p.m., in addition to 17.5% between 4.00 p.m. and 8.00 p.m., the lighting conditions were mostly good (in 89.7% of cases daylight) and the weather was usually sunny or cloudy (overall 90.8%). They mostly took place at altitudes between 500 and 1500 m (74.2%), habitually on forest roads (66.0%).

### 3.2. Characteristics of Victims

In total, 97 mountain-bikers (1 woman, 55.6 ± 13.9 years, median age 56.0 years, 1 minor) died in the Austrian Alps in the examined 16-year period. Overall, 76.3% were Austrian, 18.6% German, and 5.2% had a different nationality.

Of all documented fatalities that occurred among mountain-bikers, 54.6% of all cases had a non-traumatic origin (median age 56.0 years); among them, 96.2% were associated with cardiovascular diseases (52.6% of the total sample) and 3.8% with another disease; 41.2% of all fatalities had a traumatic origin (median age 53.5 years).

**Figure 1:**
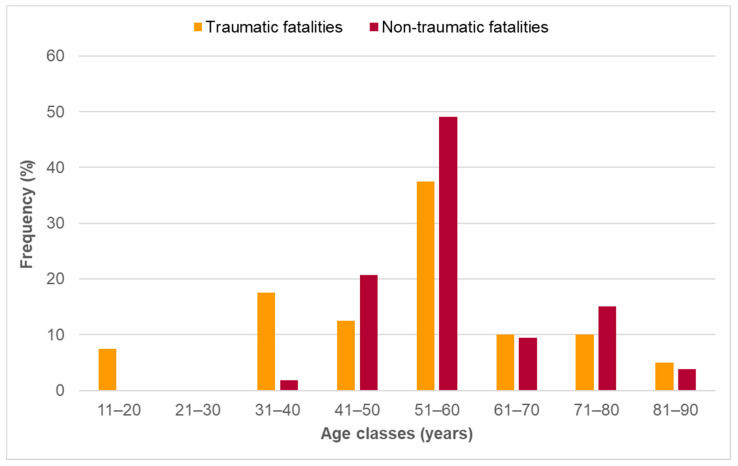
Overall cause of mortality during recreational MTB in the Austrian Alps in the period 2006–2021 divided by age class (n = 93). Orange columns = traumatic fatalities (n = 40), red columns = non-traumatic fatalities (n = 53). Values are relative frequencies. Significance level: *p* = 0.0503.

As shown in Figure 1, most of all mountain-bikers were aged between 51 and 60 years (42.3%), whereat there was a clear trend suggesting mountain-bikers accidented for non-traumatic reasons belonging more frequently to higher age classes in comparison to traumatic accidented ones (*p* = 0.05).

**Figure 2:**
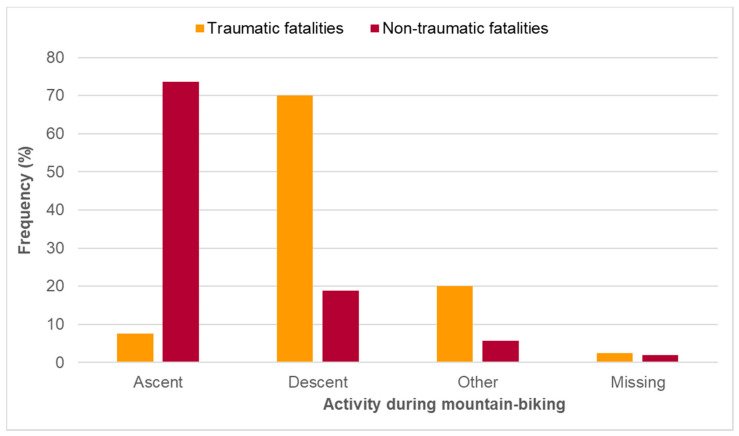
Overall cause of mortality during recreational MTB in the Austrian Alps in the period 2006–2021 divided by activity, i.e., ascent vs. descent (n = 93). Orange columns = traumatic fatalities (n = 40), red columns = non-traumatic fatalities (n = 53). Values are relative frequencies. Significance level: *p* < 0.001.

While 44.3% of fatal accidents occurred during uphill MTB, 40.2% were recorded on the way down; 13.4% happened during other activities. Figure 2 shows that the majority of traumatic fatalities (70.0%) happened during the descent, but most of the fatalities caused by non-traumatic events (73.6%) during the ascent (*p* < 0.001).

Concerning traumatic fatalities, causes of accidents, injury locations, and injury types are presented in Table 2. They were usually caused by falls on the way (40.0%) and often affected the whole body (40.0%), in which case they were classified as polytrauma.

Among the total sample, 73.2% wore a helmet, whereas 21.6% did not. There was no significant difference in helmet usage neither between victims of fatalities caused by traumatic vs. non-traumatic accidents nor concerning the type of activity (ascent vs. descent) practiced by the victims.

### 3.3. Fatal Accidents over Time and Mortality Index

**Figure 3:**
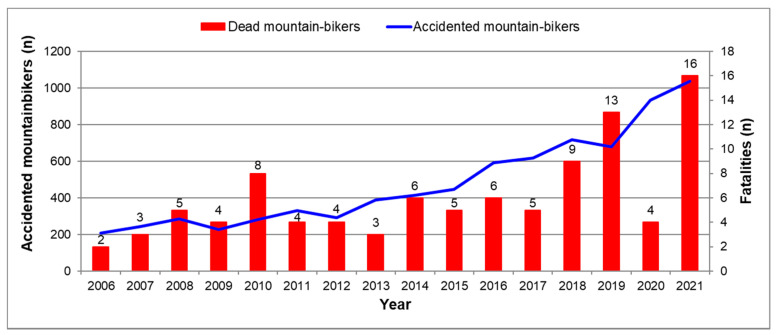
Annual number of overall fatally and non-fatally accidented (n = 7697) vs. fatally accidented (n = 97) recreational mountain-bikers in the Austrian Alps from 2006 to 2021. Values are absolute frequencies.

**Figure 4:**
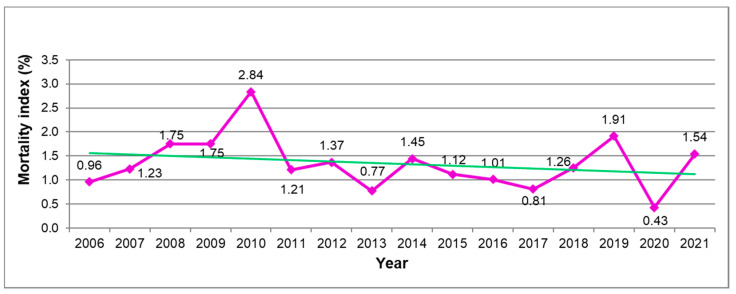
Mortality index (proportion of deaths/accidented victims) during recreational MTB in the Austrian Alps in the period between 2006 and 2021. Green line = trend line.

During the 16-year period, a significant increase in the number of overall MTB accidents, i.e., fatal and non-fatal together (*p* < 0.001), and a positive trend concerning fatal ones (*p* = 0.07) was observed (Figure 3). The resulting mortality index shows high fluctuations with a mean value of 1.34 ± 0.56% over the whole period observed, without any positive trend (Figure 4). However, the extraordinary low value of dead mountain-bikers in 2020 and consequently of the mortality index in this year, which importantly contributes to this trend, should be noted. The exclusion of the year 2020 from the regression analysis shows an increase in the number of fatalities from 2006 to 2021 (*p* < 0.001) without any significant change in the mortality index.

## 4. Discussion

The aim of the present study was to present characteristics of fatal accidents as well as resulting dead victims during recreational mountain-biking in the Austrian Alps over the past 16 years. Indeed, despite the increasing popularity of recreational MTB, there is hardly any study focusing on risks associated with the mortality related to this sporting activity [21,22]. The main results show that almost all fatalities affected males, and most of them had a non-traumatic origin, which could mainly be reconducted to cardiovascular diseases. Non-traumatic fatalities tended to affect older people frequently and occurred significantly more often during the ascent in comparison to traumatic ones, which mainly took place during the descent. Data from the past 16 years show a slight increase in the number of fatalities during mountain-biking in the Austrian Alps, although the mortality index did not increase.

### 4.1. Characteristics of Accidents

Like for fatal and non-fatal accidents during hiking in the Austrian mountains [24], also most fatalities during MTB took place in the region of Tyrol, which indeed is one of the most famous European areas for Alpine sports. As mountain-biking is a summer sport, similarly to hiking [24,26] and in line with data on MTB [18], most accidents happened in the period between May and October. In contrast to previous studies on mountain-biking conducted in Southern Alberta [18], where most severe injuries occurred during weekends compared to weekdays (61.2% vs. 38.8%), the opposite trend has been shown in the present research. It is possible that the proximity of forest ways and trails to Austrian residential areas allows a higher number of short trips also during weekdays in comparison to the Canadian environment. In line with results from the literature, most MTB accidents happened during the day, i.e., in the morning or afternoon [18], similarly as for hikers [24] in about 90% of cases with good lightening and weather conditions, defined as the sunny or clouded sky without precipitation, fog, darkness or other unfavorable circumstances [24]. Considering that about three-quarters of the victims were Austrian, it can be speculated that MTB in the Austrian Alps is mainly practiced by locals who could, similarly to Austrian skiers when compared to foreign ones [27], decide at short notice if the weather conditions are suitable for outdoor sport. Therefore, bad environmental conditions, possibly also associated with a wet surface and low or bad visibility, are probably generally avoided and seem thus not to play an important role in fatal injuries among recreational mountain-bikers in the Austrian Alps. Compared to fatal and non-fatal accidents during hiking in Austria [24], fatalities during MTB occurred mainly at a somewhat lower altitude and more often on forest roads compared to hiking accidents, which most commonly took place on marked hiking trails or small paths. These results are not surprising considering that MTB, in comparison with hiking, is mostly practiced on broader ways or paths at least uphill [8] and that these are prevalently situated in less steep mountain terrains, which are usually situated at lower altitudes.

### 4.2. Characteristics of Victims

From the whole sample, 99.0% of analyzed dead mountain-bikers were men. This is in line with previous literature that also reported a great part of severely injured mountain-bikers due to trauma in the periods 1995–2009 and 1992–2002, respectively, to have been males [18,20]. This high proportion of men among fatally injured subjects has already been shown for other alpine sports such as mountain hiking and winter sports on ski slopes [23,24,25] and could have two reasons. These are, on the one hand, the general underrepresentation of women in MTB [8,13] and, on the other hand, the potential higher risk-taking behavior of males in mountain sports activities compared with females [28]. If also sensation seeking plays a role should be assessed in future studies. There is evidence in the literature that male cyclers ride faster than female ones and that higher mean cycling speeds are associated with higher sensation-seeking scores [29]. So far, it is unclear why the proportion of fatally injured female mountain-bikers is much lower compared to that of males. It could be speculated that women die less frequently due to fewer overall MTB accidents or rather due to fewer severe MTB accidents compared to men, probably reflecting a more cautious behavior when riding a mountain-bike.

Concerning subjects’ age in the present study (55.6 ± 13.9 years, median age 56.0 years), data from previous literature show severely injured mountain-bikers having a lower median age (28 years) [18] and being more frequently part of a lower age class (21–30 years) [20] compared to the subjects of the present study. The higher age in the current research could be a result of the increasing number of sold and used e-bikes in the last years [6,7,8], which improves accessibility to MTB to older people, considering that average heart rate and perceived exertion while riding e-mountain-bikes are lower compared to riding conventional mountain-bikes [30]. Indeed, Schlemmer et al. [31] showed e-mountain-bikers spending their vacation in Tyrol (Austria) being older than tourist riders of conventional mountain-bikes. However, considering the actual median age of the Austrian population of about 43.2 years [32] and that higher age classes are represented more prominently in the present study in comparison to the overall MTB population in Austria [8], consisting mainly (52%) of mountain-bikers between 31 and 50 years (mean age: 44 years), additional causes could be hypothesized. Another contributor to this discrepancy likely is the association between higher age and sudden cardiac death during leisure time activities in the mountains [33,34].

In contrast to fatal accidents on Austrian ski slopes and to severe injuries during hiking in the Austrian Alps, which mostly concern Germans [23,24,25,35], the great part of individuals who died in the present study were Austrian mountain-bikers. This difference could indicate that MTB is not as popular as winter sports and hiking among tourists who spend their holidays in Austria.

The assessed proportion of non-traumatic deaths (54.6%) corresponds quite well to that reported for winter sports on Austrian ski slopes (48.5–52.7%) [23,25]. Like data shown for winter sports, cardiovascular events were associated with the majority (73.1–99.4%) of non-traumatic deaths [23,25]. However, compared to hiking in the Austrian mountains, where 44% of all fatalities can be reconducted to sudden cardiac death [33], cardiovascular diseases seem to be a somewhat more frequent cause of death among mountain-bikers (52.6%). It is possible that this difference is due to the higher intensity of MTB [11,36] compared to hiking, especially during the ascent, where a great part of the fatalities caused by non-traumatic events occur. Considering male sex and higher age as important risk factors for sudden cardiac death during leisure time activities in the mountains [33,34], appropriate individual health and fitness status can be considered decisive preventive factors [21].

Regarding traumatic fatalities, our results confirm evidence from the literature indicating that the most serious traumatic injuries in alpine sports happen during the descent [24,35,37]. Especially in MTB, they result from falls during downhill riding and are often caused by rapid deceleration of the bicycle leading to the rider being vaulted forward over the handlebars [38]. Future research on sports epidemiology in this recreational activity should include data on the bicycle type, i.e., conventional vs. e-mountain-bike. Indeed, considering that 89% of interviewed Austrian e-mountain-bikers started practicing this sport in the past 5 years, compared to 82% of traditional mountain-bikers, who have been practicing their leisure activity for at least 6 years [8], experience in driving downhill is supposed to influence the incidence of accidents as well as related fatal and non-fatal injuries. At this point, it is further interesting to notice the high increase in sold electrical mountain-bikes in Austria starting from the years 2017–2018 [6] and the parallel increase in the number of recorded fatalities beginning from 2018 (Figure 3).

Presented injury locations and types of fatal accidents during MTB are hardly comparable with results from the literature, as different classifications were used in research conducted in hospitals [18,20]. In particular, the items “whole body” and “polytrauma”, which were the most common injury location and type in the present research, were not present in these investigations [18,20]. However, in the current study, differently from previous ones on MTB, only fatalities have been assessed, which implies very severe injuries. On the other hand, and in line with this observation, the present rate of whole-body injuries (40.0%) is well in accordance with past research on fatally injured mountain hikers in Austria (44.3%) [24].

About ¾ of the total sample of fatally injured mountain-bikers in the present study wore a helmet. Whether severely injured mountain-bikers wore helmets are frequently poorly documented in the literature [18,20]. Previous reports assessed slightly higher rates of helmet usage of about 85% in the assessed MTB populations [20] in comparison to the present study. Nevertheless, the wearing rate of helmets was not associated with the cause of death (traumatic vs. non-traumatic) nor with the type of activity (up- vs. downhill riding) of fatally injured mountain-bikers. Taking into consideration that nearly half of traumatic deaths were classified as polytrauma, it can be speculated that fatal accidents are so severe that a helmet is not enough to ensure survival. However, considering that the use of bicycle helmets has been shown to reduce head, brain, and facial injuries [39], increasing the rate of helmet-wearing mountain-bikers still remains the highest priority.

### 4.3. Fatal Accidents over Time and Mortality Index

The mean value of the mortality index over the past 16 years (1.34 ± 0.56%) was lower compared to that shown for mountain hiking, which continuously decreased from 7.2% to 4.4% between 2006 and 2014 [24]. Similarly, also the mortality index calculated over the past 16 years for MTB showed no increase but was rather slightly declining (n.s.), being well in accordance with data from other alpine summer and winter sports practiced in Austria [24,25]. Like for mountain hiking [24], this development may indicate that slightly rising absolute numbers of fatal MTB accidents do not reflect that MTB becomes more dangerous but rather is related to the rising popularity of MTB [8]. However, while the absolute frequency of dead hikers has remained stable over 9 years [24], the number of fatal accidented mountain-bikers has moderately increased during the past 16 years.

Even if single values of the mortality index should not be overvalued, as they are influenced by a small number of fatalities per year, the all-time low value of the mortality index in 2020 seems to be remarkable. As shown in Figure 3, this outstanding rate in 2020 is primarily influenced by the low number of fatally injured mountain-bikers. Indeed, the constant rising trend over time in the absolute frequency of overall accidented riders, which also continues in 2020, does not give any indication of a changing trend concerning the number of mountain-bikers in the Austrian Alps. Since 2020 was the year of the beginning of the COVID-19 pandemic, it is possible that people practicing leisure sporting activities in the Austrian Alps in 2020 were more cautious to avoid intensive care units, which were in large part occupied by COVID-19 patients. This is in line with the opinion expressed by Posch et al. [40]. Moreover, like for alpine skiing on Austrian mountains [40], for the same reason, high-risk subjects for non-traumatic (i.e., cardiovascular) events were expected to be more cautious and therefore reduce or abstain from intensive sport activities. Some evidence for these hypotheses is given by van Aert et al. [41], who showed a significant decrease in sports-related injuries during the lockdown in 2020 compared to 2018 and 2019. If a possible change in the composition of the overall MTB population in the Austrian Alps in 2020 (because of the travel restrictions for tourists) could have had an influence on the mortality index in 2020 remains unclear. However, considering that locals rated their behavior on Austrian ski slopes more frequently as risky compared to foreign guests [27], this assumption seems not to be supposable.

### 4.4. Suggestions for the Prevention of Fatal Accidents

Although fatal events during mountain biking are relatively rare, specific preventive efforts are needed to further reduce such incidents. Regarding non-traumatic deaths, regular (intense) physical activities, as known from other mountain sports activities, may be helpful through conditioning the cardiovascular and respiratory systems [33,42,43]. Concerning traumatic fatalities, awareness of the fall risk during downhill riding, improvements in safety equipment, and appropriate rider training (especially for beginners and e-mountain-bikers) will likely help to avoid some of the most severe traumatic events [38,44].

### 4.5. Limitations

Some limitations should be pointed out concerning this study. First, data were collected by the Alpine Police, which has other priorities than scientific research. Therefore, information was limited for organizational reasons and did not include parameters that could have an important influence on research in this field, e.g., information on the type of bike used (conventional vs. e-mountain-bike). On the other hand, this procedure ensures highly standardized data input by well-trained staff [24]. Moreover, no medical diagnoses were available. Therefore, we suggest future research trying to link institutional data with medical information.

The low number of fatalities per year should be considered, especially for the interpretation of the mortality index. Nevertheless, the calculation over a 16-year period relativizes possible outliners. Furthermore, as reported for mountain hikers [24], also for mountain-bikers, the aforementioned index can only be calculated using the number of accidented subjects as a collectivity. Indeed, in contrast to other sports, especially alpine skiing, for which, for example, data on skier days are available, neither information on the exact number of mountain-bikers in the Austrian Alps nor on their exposure time can be provided. However, the present research is one of the first investigations pointing out some factors associated with mortality in MTB.

## 5. Conclusions

The current research is among the first studies characterizing in detail mortality in recreational MTB. The present results identify some factors associated with mortality in MTB in the population of interest, including male subjects being at higher risk for fatal events compared to women. In particular, older mountain-bikers are more exposed to non-traumatic, especially cardio-vascular events during the ascent; in contrast, traumatic events are more frequent in downhill riding. Therefore, primarily for male mountain-bikers older than 40 years, possible health-related risk factors, including those of cardiovascular nature, should be checked and considered regarding the level of effort and exhaustion. On the other hand, more attention should be put on the risk of traumatic events during the descent, especially among male riders. Preventive measures in order to reduce fatal events in recreational MTB seem to be of utmost importance, especially if we consider that absolute frequencies of deaths are slightly increasing over the past years. Therefore, especially male riders could be targeted by public health strategies aiming for a reduction of traumatic and non-traumatic accidents.

## Figures and Tables

**Table 1:** Environmental characteristics associated with fatal accidents of recreational mountain-bikers in the Austrian Alps during the period 2006–2021. Data are presented as absolute and relative frequencies.

	Frequency	
	(n)	(%)
**Region** (n = 97)		
Burgenland	0	0.0
Carinthia	6	6.2
Lower Austria	8	8.2
Salzburg	19	19.6
Styria	12	12.4
Tyrol	34	35.1
Upper Austria	13	13.4
Vienna	1	1.0
Vorarlberg	4	4.1
**Time of year: month** (n = 97)		
March	2	2.1
April	5	5.2
May	11	11.3
June	25	25.8
July	11	11.3
August	16	16.5
September	17	17.5
October	10	10.3
**Time of week** (n = 97)		
Weekday	59	60.8
Weekend	38	39.2
**Time of day** (n = 97)		
Early morning (12:01 a.m.–8:00 a.m.)	1	1.0
Morning/afternoon (8:01 a.m.–04:00 p.m.)	77	79.4
Evening (4:01 p.m.–8:00 p.m.)	17	17.5
Night (8:01 p.m.–12:00 a.m.)	2	2.1
**Lighting conditions** (n = 92)		
Daylight	87	94.6
Twilight	1	1.1
Darkness	4	4.3
**Weather conditions** (n = 93)		
Sunny	76	81.7
Cloudy	12	12.9
Rainy	4	4.3
Snowy	1	1.1
**Altitude** (n = 89)		
<500 m	3	3.4
500–1000 m	33	37.1
1000–1500 m	39	43.8
1500–2000 m	14	15.7
**Type of trail** (n = 89)		
Asphalt road	1	1.1
Drive or forest way	64	71.9
Marked hiking trail or small path	13	14.6
Pathless terrain	7	7.9
Bike-park	2	2.2
Other	2	2.2

**Table 2:** Causes of accident, injury locations, and injury types associated with traumatic fatal deaths of recreational mountain-bikers (n = 40) in Austria during the period 2006–2021. Data are presented as absolute and relative frequencies.

	Frequency	
	(n)	(%)
**Cause of accident**		
Fall on the way	16	40.0
Fall down hillside	12	30.0
Fall in a creek/river	10	25.0
Collision	2	5.0
**Injury location**		
Head	7	17.5
Neck/cervical spine (CS)	6	15.0
Back/spine without CS	4	10.0
Whole body	16	40.0
Missing	7	17.5
**Injury type**		
Fracture	11	27.5
Luxation	1	2.5
Internal injury	2	5.0
Polytrauma	19	47.5
Missing	7	17.5

## Data Availability

Data sets are stored by the Austrian Board for Alpine Safety (ÖKAS), https://alpinesicherheit.at (accessed on 13 June 2022).
